# Comparison between Different Intensity Normalization Methods in ^123^I-Ioflupane Imaging for the Automatic Detection of Parkinsonism

**DOI:** 10.1371/journal.pone.0130274

**Published:** 2015-06-18

**Authors:** A. Brahim, J. Ramírez, J. M. Górriz, L. Khedher, D. Salas-Gonzalez

**Affiliations:** Department of Signal Theory, Networking and Communications, University of Granada, Granada, Spain; University of Verona, ITALY

## Abstract

Intensity normalization is an important pre-processing step in the study and analysis of DaTSCAN SPECT imaging. As most automatic supervised image segmentation and classification methods base their assumptions regarding the intensity distributions on a standardized intensity range, intensity normalization takes on a very significant role. In this work, a comparison between different novel intensity normalization methods is presented. These proposed methodologies are based on Gaussian Mixture Model (GMM) image filtering and mean-squared error (MSE) optimization. The GMM-based image filtering method is achieved according to a probability threshold that removes the clusters whose likelihood are negligible in the non-specific regions. The MSE optimization method consists of a linear transformation that is obtained by minimizing the MSE in the non-specific region between the intensity normalized image and the template. The proposed intensity normalization methods are compared to: *i*) a standard approach based on the specific-to-non-specific binding ratio that is widely used, and *ii*) a linear approach based on the α-stable distribution. This comparison is performed on a DaTSCAN image database comprising analysis and classification stages for the development of a computer aided diagnosis (CAD) system for Parkinsonian syndrome (PS) detection. In addition, these proposed methods correct spatially varying artifacts that modulate the intensity of the images. Finally, using the leave-one-out cross-validation technique over these two approaches, the system achieves results up to a 92.91% of accuracy, 94.64% of sensitivity and 92.65 % of specificity, outperforming previous approaches based on a standard and a linear approach, which are used as a reference. The use of advanced intensity normalization techniques, such as the GMM-based image filtering and the MSE optimization improves the diagnosis of PS.

## Introduction

Parkinsonian syndrome (PS) or Parkinsonism is characterized by the presence of hypokinesia associated with rest tremor and/or rigidity and/or postural instability. From a clinical point of view, the most common condition in this syndrome is Parkinson’s disease (PD). PD is a severe progressive neurodegenerative disorder which is neuropathologically characterised by the progressive loss of dopaminergic neurons of the nigrostriatal pathway. This leads to a corresponding loss of dopamine transporters (DaTs) in the striatum [1]. The DaTs are proteins situated at the presynaptic terminal of dopaminergic neurons which are responsible for the re-uptake of dopamine. In order to visualize the loss of dopamine by means of brain imaging techniques, such as single-photon emission computed tomography (SPECT) or positron emission tomography (PET), different radio-ligands can be used, such as I-Ioflupane (better known as DaTSCAN [2, 3] or [^123^I]FP-CIT [4]), [^123^I]-*β*-CIT, [^123^I]IBZM [5] and [Tc-99m]-TRODAT-1 [6] which binds to the dopamine transporters in the striatum. The standard method for analyzing DaTSCAN SPECT images is by calculation of the binding potential (BP) in the striatum. BP is a quantitative measure of specific tracer binding, and is lower in PD patients as compared to healthy subjects [7, 8]. This specific-to-non-specific binding ratio can then be estimated as [9]:

$$BP=\frac{C_{VOI}-C_{N}}{C_{N}}=\frac{C_{VOI}}{C_{N}}-1$$ (1)

where **C**_*VOI*_ is the mean count per voxel in the volume of interest (striatum, putamen or caudate nucleus) and **C**_*N*_ represent the mean count per voxel in the non-specific binding region (occipital cortex). This binding ratio is widely used in the literature for normalization purpose in different functional brain images [10–12], such as DaTSCAN SPECT images. The occipital region was chosen as a reference region because of the negligible density of DaTs [13]. For this purpose, clinicians often use proprietary software to delimit regions of interest (ROIs) and quantify the radiopharmaceutical uptake [14]. This procedure can be subjective and prone to error, since it relies on gross changes in transporter density throughout the ROIs to allow the differentiation between controls and pathological images. As such, it may not be sensitive to changes in the pattern of distribution that can characterize the progression of the disease [15]. In contrast, some more automatized methods have been proposed which establishes semi-quantitative parameters in order to index absolute differences between specific/non-specific uptake in the tomographic examinations [16]. For this purpose, it is necessary that the images are quantitative, in the sense that the image value at each voxel is proportional to the activity concentration. The quality of acquired images is degraded by both physical factors, such as compton scattering and photon attenuation, and system parameters, such as intrinsic and extrinsic spatial resolution of the gamma camera system. These factors result in blurred and noisy images. Most times, the blurred images present artifacts that may lead to a fault diagnosis. In order to gain a fair diagnostic of the DaTSCAN imaging for the physician, it is compulsory to follow a specific series of processing, such as, scattering and attenuation correction during the image reconstruction procedure, and preferably also resolution compensation or partial volume correction [17]. In addition, the intensity normalization is a relevant preprocessing step, which guarantees that the differences between images of different subjects are due to physiological reasons and brain functioning, and not due to the baseline calibration of the gamma camera applied for the acquisition [18]. The conventional way of carrying out the intensity normalization is to consider as a reference the brain region which is not significant as a differentiating criterion between, both ill and healthy image subjects. Since the discriminant region for PD is the striatum, the occipital region is usually chosen as a reference because it is devoid of DATs and it is usually selected as the background region. However, in this work, the whole brain area is considered, minus the striatum, as a non-specific region [19]. The main reasons for this choice are that DaTSCAN images contain considerably fewer anatomic details and omit structural details about the location of the occipital cortex (the normalization region). Furthermore, the partial volume effect causes blurring of counts from the grey matter into the ventricular space, often to such an extent that the ventricles are practically indistinguishable, sometimes making it difficult to use confidently occipital or frontal cortex regions. The proposed use of the overall non-specific region should reduce variability as well as improve counting statistics [14]. Thus, this image preprocessing stage consists of comparing the uptake value in areas of specific activity (binding to dopaminergic transporters) to the value in areas of non-specific activity (vascular activity) between subjects. The current normalization methods, such as specific-to-non-specific binding ratio, depends on time consuming operator-intensive work, expertise skills in manually placing the regions of interest (ROI) and it use the mean intensity value in the so-defined non-specific region as a reference.

This paper shows two novel methods of automatic intensity normalization of DaTSCAN SPECT images using GMM-based image filtering and MSE optimization approaches that eliminate operator-dependent manipulations [20]. In this sense, the proposed methods do not require the manual pre-selection of relevant information by means of statistical analysis [9]. The main novelty with respect to previous approaches [21] is that these proposed methods work spatially and locally on the image in order to automatically normalize the reference region. Moreover, filtering by means of GMM based strategy allows us to *modulate or filter* the voxel intensity by discarding the clusters whose probability is below a normalized probability threshold in the specific region (striatum). We consider these deleted Gaussians with the low intensity profile as a reference region because most of these irrelevant clusters are located in the occipital cortex. On the other hand, MSE optimization performs a linear transformation of the intensity values by estimating the different intensity normalization parameters that leads to minimize the MSE between the intensity normalized image and the template. Thus, GMM-based image filtering and MSE optimization approaches are used as a filtering and normalizing strategies to remove artifacts and noise, such as Gaussian noise [22, 23] after the image acquisition stage. These proposed methods are evaluated not only in terms of processing and analyzing SPECT image data but also in the classification performance for improving the diagnostic accuracy in Parkinsonism. All the proposed methods were implemented using Matlab software, as well as, the experiments carried out to evaluate them.

## Materials and Methods

### Ethics Statement

This database is derived from nuclear medicine departments at “Virgen de la Victoria” public hospital in Malaga (Spain) [24] where the participants provided their written informed consent. The ethics committee of this hospital approved the former studies, respectively.

### DaTSCAN SPECT dataset

**Fig 1:**
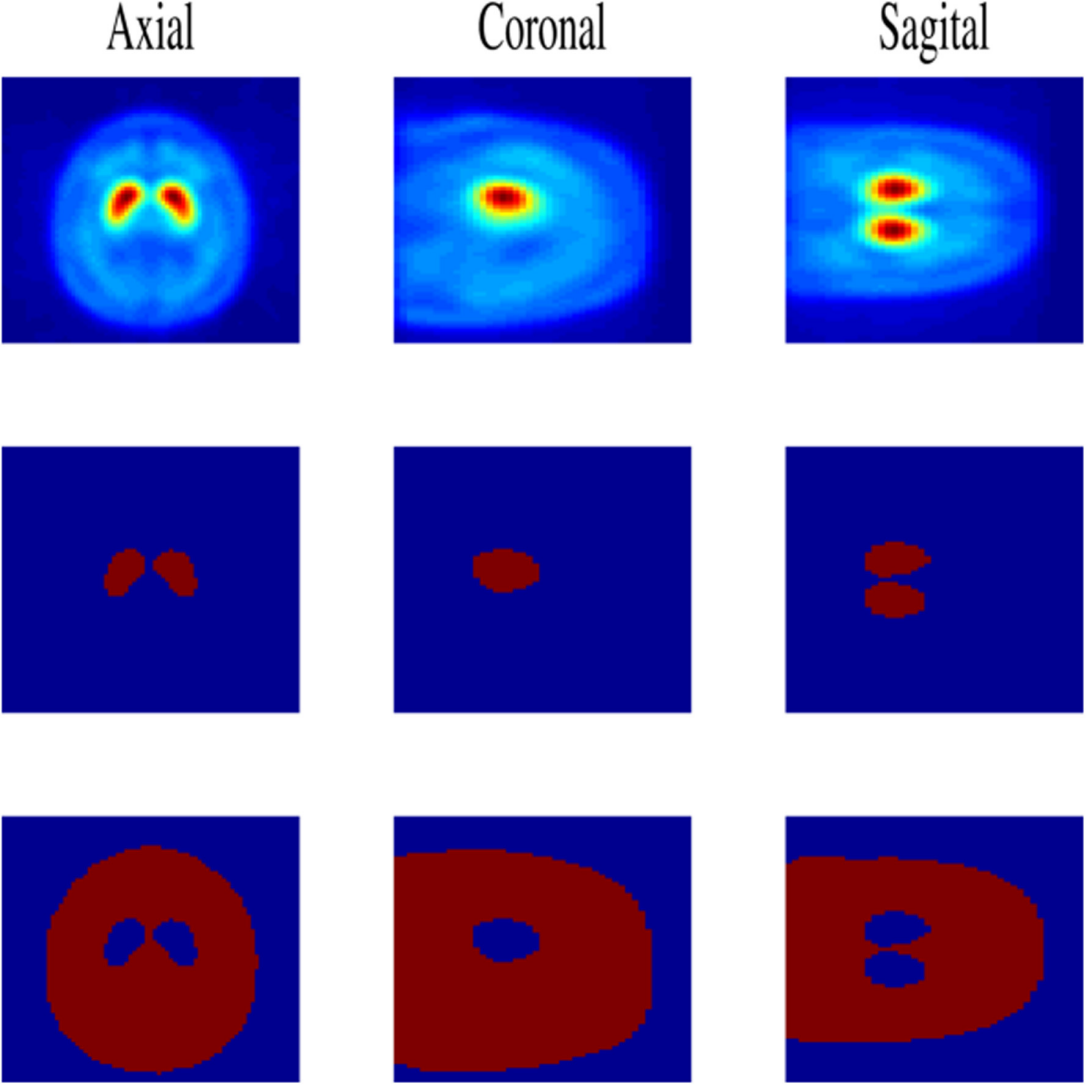
The computed template and the different masks used in the intensity normalization process. First row: template image generated by averaging the NCs. Second row: the striatum mask applied in FGMM approach. Third row: the non-specific mask applied in MSE approach.

To evaluate the proposed methodologies a database consisting of 189 SPECT images from 189 subjects (94 Normal Controls (NCs) and 95 Parkinsonian Syndrome (PS)), was obtained after the injection of 185 MBq (5 mCi) of the radioligand: Ioflupane-I-123 after an extension of time between 3–4 h; during this period, the thyroid was blocked using a Lugols solution. The SPECT images with Ioflupane/123I-FP-CIT were obtained by a using a General Electric gamma camera, Millennium model, equipped with a dual head and general purpose collimator. A 360-degree circular orbit was made around the cranium, at 3-degree intervals, leading to 60 images each 35 seconds per interval and with 128 × 128 matrix. The brain images were reconstructed using the filtered back projection algorithm, applying a Hanning filter (cut-off frequency equal to 0.7) and were obtained with transaxial slices. To avoid variability from additional image processing, no attenuation or scatter correction was applied in this study. Those images were acquired by the “Virgen de la Victoria” hospital from January 2003 until December 2008 (see Table 1 for demographic details). All the SPECT images were spatially normalized using the SPM 8 software [25] yielding a 73 × 73 × 45 three-dimensional functional activity map for each subject. This method assumes a general affine model with 12 parameters and a Bayesian framework that maximizes the product of the prior function (which is based on the probability of obtaining a particular set of zooms and shears) and the likelihood function, derived from the residual squared difference between the template and the processed image. The template *t* is computed by registering all control images to a randomly chosen one by affine transformations. This *N*_*c*_ = 94 controls and its hemisphere midplane reflected that the images are averaged to create the template [26], providing a symmetric image, as shown in the first row of the Fig 1.

$$t=\frac{1}{N_{c}}\sum_{i\in X_{c}}\left(I_{i}\left(x,y,z\right)+I_{i}\left(-x,y,z\right)\right)$$ (2)

where *X*_*c*_ denotes the subset of control images, *N*_*c*_ the number of control images, *I*_*i*_(*x*, *y*, *z*) is the ith image and *I*_*i*_(−*x*, *y*, *z*) is its reflected image in the *x* = 0 hemisphere midplane. The main reasons of building the template *t* by a simple averaging process of co-registered images from healthy patients [27, 28] are that DaTSCAN SPECT images provide low resolution smoothed functional maps about the uptake in the striatum area with limited morphological information. In addition, intensity normalization is aimed to correct inter-subject variability in the intensity level of the image due to a variety of reasons related to the acquisition process. Thus, high resolution morphological information is not required by intensity normalization since the algorithms are often based on descriptive statistics related to the specific and/or non-specific areas to correct global variations of the intensity. However, there are many developed methods to build the template for MRI where the morphological changes in tissues are important for most of the applications, such as the mean shape method [29, 30].

**Table 1:** Demographic details of the subjects who participated in this study. *μ* and *σ* stand for the average and the standard deviation respectively.

		Sex		Age		
	#	M	F	*μ*	*σ*	range
NCs	94	49	45	69.26	10.16	33–89
PS	95	54	41	68.29	9.62	30–87

This spatial normalization ensures that any given voxel in different images refers to the same anatomical position across the brains. Once the images have been properly normalized, they were visually labeled by three nuclear medicine specialists from the hospital using only the information contained in the images, without any other medical information [31]. The assessments were done without trying to assign them to different clinical groups within the set of pathological studies. A study was considered to be normal when bilateral, symmetrical uptake appeared in caudate and putamen nuclei, and abnormal when there were areas of qualitative reduced uptake in any of the striatal structures.

### Filtering by means of GMM (FGMM)

The GMM is an efficient method for classification and density estimation [32–34] and can modulate the intensity in any position of the image [35] according to this equation:

$$I_{Gauss}\left(x\right)=I\cdot p\left(x\right)$$ (3)

where *I* is the total intensity of the image and *p*(**x**) is the probability distribution for a spatial coordinate **x**, which is modeled by a sum of *k* Gaussians [36]:

$$p\left(x\right)=\sum_{n=1}^{k}w_{n}f_{n}\left(x|\theta_{n}\right)$$ (4)

where *f*_*n*_(**x**|*θ*_*n*_) is the density of the *n*-th Gaussian with parameter vector *θ*_*n*_ and *w*_*n*_ are the weight factors or mixing proportions with ∑_*n*_ *w*_*n*_ = 1. The number of clusters is selected according to an information criterion for model selection, such as the one based on the minimization of the MSE between the original and the GMM-reconstructed images as shown in [37]. In this way, a number of clusters *k* = 64 in Eq 4, is suitable for achieving a trade-off between error reconstruction and dimension reduction (related to the model adjustment). In fact, the selection of a larger number of Gaussians will vastly increase the computational cost [38]. This probability model can be used to increase the difference in intensity between the specific and non-specific areas in DaTSCAN imaging, that is, to enhance signal to noise ratio (SNR). In this sense, an intensity normalization procedure based on cluster prunning is shown in the following:

- First, a striatal mask is computed by thresholding the average image of NCs, as shown in the section below, in which the ROI selection is described. In the second row of Fig 1, the resulting mask is shown. Then, the coordinates of each voxel belonging to the striatum, denoted by $\text{SR}=\{x_{j}^{s}\}$ for *j* = {1, …, *N*_*s*_}, are selected, where *N*_*s*_ is the number of voxels within the striatum mask.
- Second, a cluster selection strategy is applied in the spatial domain in order to automatically select the relevant clusters that contribute the SR region. For this purpose, a normalized probability threshold *η* is defined in order to preserve the intensity in this *key* area. If the total intensity is assumed to be uniformly distributed in the image, the probability of each coordinate is *p*_*u*_(**x_j_**) = 1/*N* ≡ *η* ≅ 4⋅10^−6^, where *N* is the total number of voxels. Thus, the conditional probability value of a given voxel in SR that satisfies:
  $$f_{n}\left(x_{j}|\theta_{n}\right)<\eta \;n=1,\ldots ,k$$ (5)
  reflects a deviation from the uniform threshold value, that is, a negligible contribution to Eq 4 evaluated on this particular voxel. A cluster is considered as irrelevant in SR region if this inequality holds for a large fraction of *N*_*s*_, i.e. 75%. This cluster is removed from the mixture model (Eq 4) by setting its weight *w*_*n*_ to zero, as a result, the remaining clusters are conserved and used for the filtered image reconstruction.

**Fig 2:**
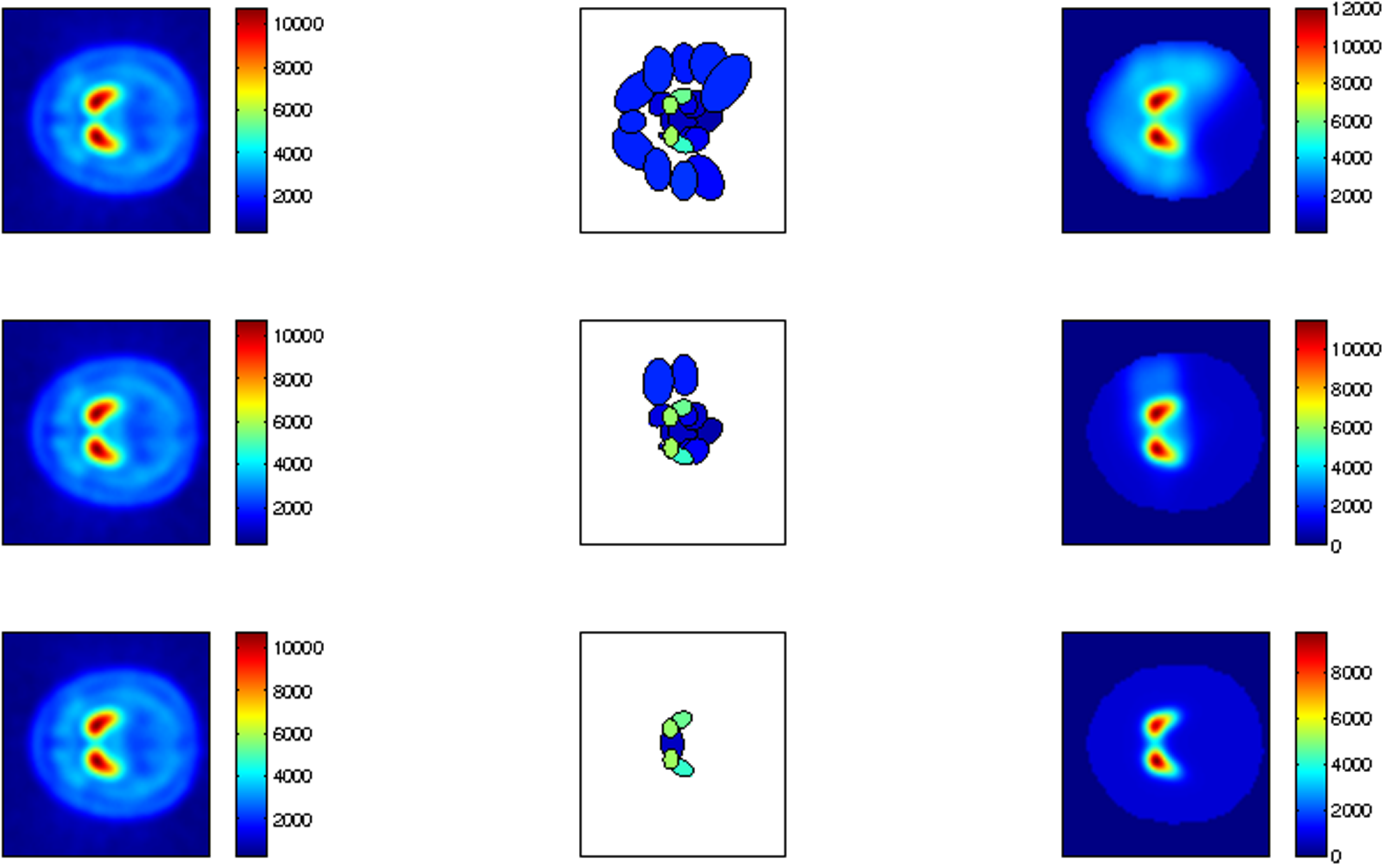
Filtered GMM average image according to different probability thresholds. Left column: DaTSCAN image of average normal subjects. Central column: different location and intensity of relevant clusters for different values of threshold ***η*** = 4 ⋅ 10^−6^, 4 ⋅ 10^−5^ and 4 ⋅ 10^−4^. Right column: filtered GMM image reconstruction according to Eq 5.

Several values for the fraction of *N*_*s*_ to define cluster relevance were tested, i.e. 75% with the probability threshold equal to the uniform value, as shown in the first row of Fig 2. According to this procedure, a filtered GMM image reconstruction is achieved that: *i*) preserves the intensity in the specific region and *ii*) automatically normalizes the intensity in the non-specific areas (Fig 2) in such a way that the inter-subject intensity differences are reduced as shown in the experimental part.

The first row of the Fig 2 depicts the filtered GMM average image (right column) according to a probability threshold equal to the uniform value (***η*** = 4⋅10^−6^), which preserves the shape and the intensity of the striatal signal. The remaining rows exhibit the effect of another probability thresholds (4⋅10^−5^ and 4⋅10^−4^) on the intensity normalization in the specific and non-specific regions. For instance, in the right column of the third row, the shape of the striatum is clearly affected. Left and central columns depict, respectively, the DaTSCAN image of average normal subjects and the different locations and intensities of relevant clusters for different values of probability threshold.

### Mean Squared Error Optimization

**Fig 3:**
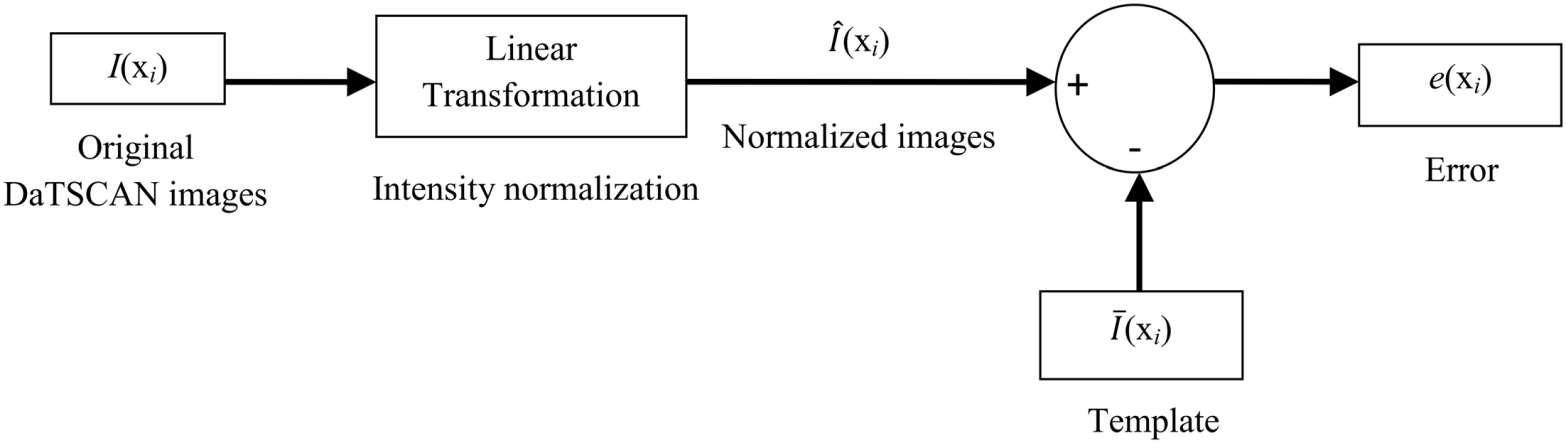
General diagram of linear intensity normalization method for DaTSCAN SPECT images using the MSE approach.

The MSE is widely used as a metric for quality assessment of medical image [39]. In this work, its minimization can involve a novel intensity normalization method for DaTSCAN SPECT images. To state the problem, let *I*(**x**_*i*_), $\bar{I}({\text{x}}_{i})$ and $\hat{I}({\text{x}}_{i})$ denote the intensity values of the original, template and normalized images in the non-specific region (Fig 3). In MSE optimization, an estimate $\hat{I}({\text{x}}_{i})$ is to be found that minimizes the cost function *ξ*:

$$\xi =\frac{1}{N_{ns}}\sum_{i=1}^{N_{ns}}{|\hat{I}\left(x_{i}\right)-\bar{I}\left(x_{i}\right)|}^{2}$$ (6)

where *N*_*ns*_ is the number of voxels in the non-specific region. Although the solution to this problem generally leads to a nonlinear estimator, in many cases a linear estimator is preferred [40]. In linear mean-square estimation, we assume that the image intensity levels are related by the following model:

$$\hat{I}\left(x_{i}\right)=a\;I\left(x_{i}\right)+b$$ (7)

where *a* and *b* are the intensity normalization parameters, they represent the scale and offset of the intensity transformation [41]. The aim of MSE optimization, is to linearly transform the intensity heterogeneity in the non-specific region for different DaTSCAN SPECT images by jointly estimating the parameters *a* and *b* in Eq 7. This leads to the joint minimization of a cost function *ξ* that is expressed as:

$$\xi =\frac{1}{N_{ns}}\sum_{i=1}^{N_{ns}}e^{2}\left(x_{i}\right)$$ (8)

where e(**x**_*i*_) is the estimation error, namely

$$e\left(x_{i}\right)=\hat{I}\left(x_{i}\right)-\bar{I}\left(x_{i}\right)=a\;I\left(x_{i}\right)+b-\bar{I}\left(x_{i}\right)$$ (9)

Solving the linear mean-square estimation problem may be accomplished by differentiating *ξ* with respect to *a* and *b* and setting the derivatives equal to zero as follows:

$$\frac{\partial \xi }{\partial a}=\frac{1}{N_{ns}}\sum_{i=1}^{N_{ns}}\frac{\partial e^{2}\left(x_{i}\right)}{\partial a}=\frac{1}{N_{ns}}\sum_{i=1}^{N_{ns}}2\;e\left(x_{i}\right)\;I\left(x_{i}\right)=0$$ (10)

$$\frac{\partial \xi }{\partial b}=\frac{1}{N_{ns}}\sum_{i=1}^{N_{ns}}\frac{\partial e^{2}\left(x_{i}\right)}{\partial b}=\frac{1}{N_{ns}}\sum_{i=1}^{N_{ns}}2\;e\left(x_{i}\right)=0$$ (11)

Note that Eq 10 is the orthogonality principle [40] and states that for the optimum linear predictor the estimation error will be orthogonal to the data *I*(**x**_*i*_). From Eqs 7, 9, 10 and 11, it follows that

$$a\sum_{i=1}^{N_{ns}}I^{2}\left(x_{i}\right)+b\sum_{i=1}^{N_{ns}}I\left(x_{i}\right)-\sum_{i=1}^{N_{ns}}\bar{I}\left(x_{i}\right)I\left(x_{i}\right)=0$$ (12)

$$a\sum_{i=1}^{N_{ns}}I\left(x_{i}\right)+b\;N_{ns}-\sum_{i=1}^{N_{ns}}\bar{I}\left(x_{i}\right)=0$$ (13)

Solving Eqs 12 and 13 for *a* and *b* we find:

$$a=\frac{\sum_{i=1}^{N_{ns}}I\left(x_{i}\right)\bar{I}\left(x_{i}\right)-N_{ns}\;m_{\bar{I}}\;m_{I}}{\sum_{i=1}^{N_{ns}}I^{2}\left(x_{i}\right)-N_{ns}\;m_{I}^{2}}$$ (14)

$$b=m_{\bar{I}}-a\;m_{I}$$ (15)

where

$$m_{I}=\frac{1}{N_{ns}}\sum_{i=1}^{N_{ns}}I\left(x_{i}\right);\;m_{\bar{I}}=\frac{1}{N_{ns}}\sum_{i=1}^{N_{ns}}\bar{I}\left(x_{i}\right)$$ (16)

Substituting Eq 15 into Eq 7, the estimate for $\hat{I}({\text{x}}_{i})$ may be written as:

$$\hat{I}\left(x_{i}\right)=a\;I\left(x_{i}\right)+\left(m_{\bar{I}}-a\;m_{I}\right)=a\;\left(I\left(x_{i}\right)-m_{I}\right)+m_{\bar{I}}$$ (17)

As a result, the normalized image $\hat{I}({\text{x}}_{i})$ can be expressed according to the original DaTSCAN image *I*(**x**_*i*_) and the intensity normalization parameters as:

$$\hat{I}\left(x_{i}\right)=\frac{\sum_{i=1}^{N_{ns}}I(x_{i})\bar{I}\left(x_{i}\right)-N_{ns}\;m_{\bar{I}}\;m_{I}}{\sum_{i=1}^{N_{ns}}I^{2}\left(x_{i}\right)-N_{ns}\;m_{I}^{2}}\;\left(I\left(x_{i}\right)-m_{I}\right)+m_{\bar{I}}$$ (18)

After obtaining the optimum linear estimator for $\hat{I}({\text{x}}_{i})$, the minimum MSE can be evaluated as:

$$\begin{array}{c} \begin{array}{c} \xi_{\text{min}}=\frac{1}{N_{ns}}\sum_{i=1}^{N_{ns}}e\left(x_{i}\right)\;\left(a\;I\left(x_{i}\right)+b-\bar{I}\left(x_{i}\right)\right)=-\frac{1}{N_{ns}}\sum_{i=1}^{N_{ns}}e\left(x_{i}\right)\;\bar{I}\left(x_{i}\right)\\ =\frac{1}{N_{ns}}\;\left(\;\sum_{i=1}^{N_{ns}}{\bar{I}}^{2}\left(x_{i}\right)-\;b\sum_{i=1}^{N_{ns}}\bar{I}\left(x_{i}\right)-a\sum_{i=1}^{N_{ns}}\;I\left(x_{i}\right)\bar{I}\left(x_{i}\right)\;\right) \end{array} \end{array}$$ (19)

In summary, this intensity normalization procedure for DaTSCAN SPECT images is outlined as:

- Firstly, a non-specific mask is computed as the difference between the skull and the striatum masks of the template in a binary form, as shown in the third row of Fig 1. Then, it is applied to all images in order to select the brain voxels minus the striatum as non-specific region.
- Secondly, the average intensity of brain voxels **m**_*I*_ and $\;m_{\bar{I}}$ are computed for the source *I*(**x**_*i*_) and the template $\bar{I}({\text{x}}_{i})$ images in the non-specific areas.
- Lastly, the intensity normalization parameters a and b are calculated using Eqs 14 and 15. As a result, a linear intensity transformation is applied to each source image that minimizes the MSE between the latter image and the template.

## Results and Discussion

### Qualitative analysis

**Fig 4:**
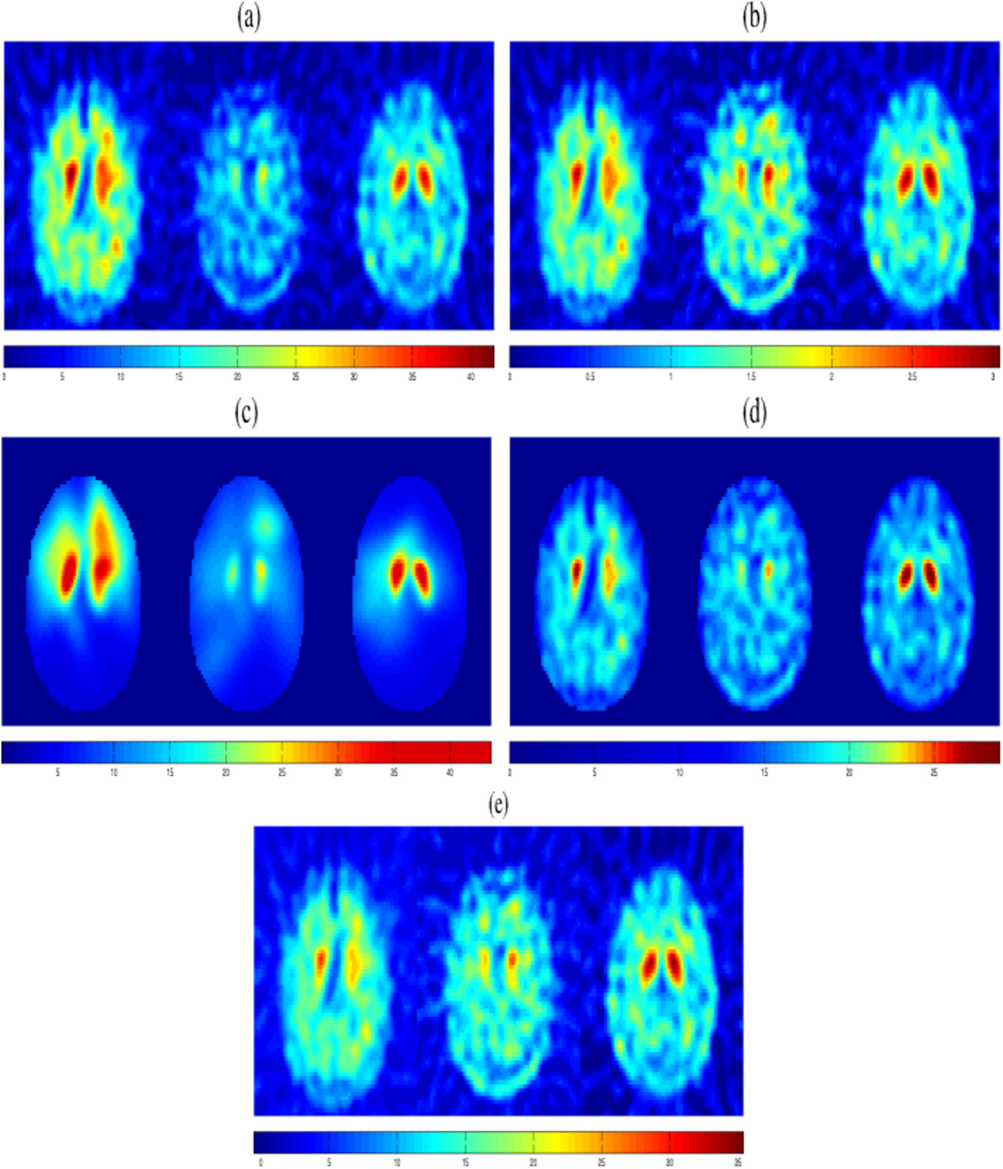
A given transaxial slices of 3 selected brain images before and after intensity normalization. (a) original DaTSCAN images from the database (Raw data), (b) normalized brain images by specific-to-non-specific ratio approach (BR_*all*_ images), (c) normalized brain images by filtering by means of GMM approach (FGMM images), (d) normalized brain images by MSE approach (MSE images) and (e) normalized brain images by *α*-stable approach (*α*-stable images).

The proposed methodologies have been tested using 127 different DaTSCAN images (68 NCs and 59 PS subjects) from the database described in DaTSCAN SPECT dataset subsection which presents a high degree of variability of the intensity level for the specific/non-specific area, as can be seen in Fig 4a. Furthermore, these images present a relatively low SNR in the non-specific region provided by the image acquisition system in the nuclear medicine department. A visual inspection of the mean histograms of the raw data suggests that this variability is not produced by a multiplicative parameter in the data [21]. Therefore, a normalization procedure using only a bias, as the specific to non-specific binding ratio (BR_*all*_) [42], is not enough for an accurate intensity normalization procedure as it affects the shape and the intensity of the striatal signal (Fig 4b). BR_*all*_ denotes the binding ratio calculated using all the brain voxels, except those in the striatum, as non-specific region. In this work, it is used as a baseline. By applying the two proposed intensity normalization methods detailed in the methodological sections, the intensity heterogeneity in the non-specific region is reduced and the difference between the striatum and the background uptakes is increased as shown in Fig 4c and 4d. These figures demonstrate that the inter-subject intensity differences in the non-specific region due to several effects [43–45] are clearly reduced after normalization. Unlike those ones shown in Fig 4b and 4e, the processed images are smooth and preserve the relevant information in the striatum region. In addition, the proposed normalization approaches allow us to guarantee that the inter-subject differences in the DaTSCAN image database (NC and PS subjects) are due only to the uptake of the tracer in the discriminant region (striatum) and not due to the baseline calibration of the gamma camera used for the acquisition.

### Quantitative analysis

**Fig 5:**
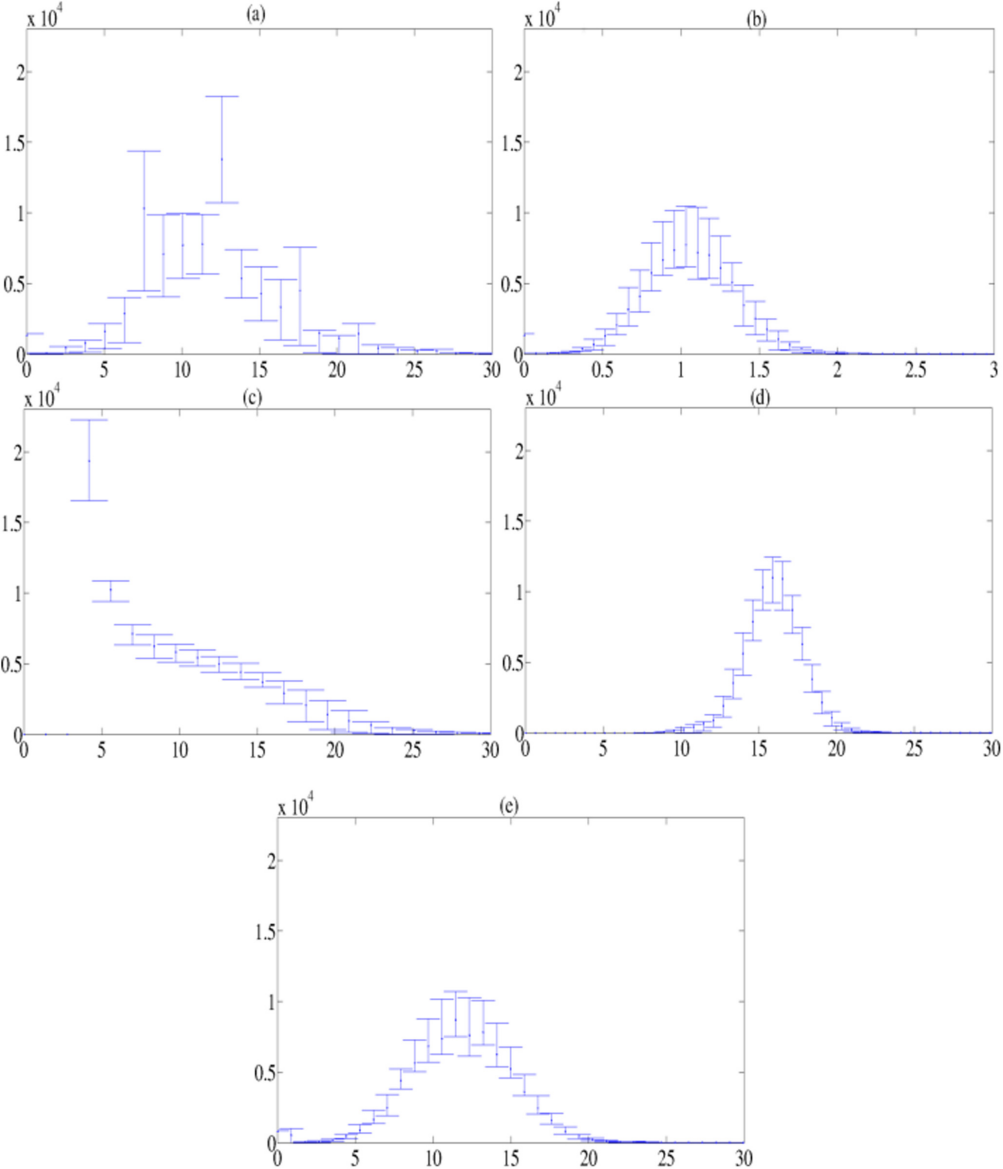
Mean histograms and error bars in the non-specific region for 127 DaTSCAN images before and after intensity normalization. (a): Original DaTSCAN images, (b): BR_*all*_ images, (c): FGMM images, (d): MSE images and (e) *α*-stable images. The x-axis represents the intensity. The y-axis indicates the number of voxels with a given level in the non-specific region.

In order to quantitatively measure the efficiency of the proposed intensity normalization methods, we compare them with the original DaTSCAN brain images (before intensity normalization) and another normalization methods widely used which are the intensity normalization by BR_*all*_ and the linear intensity normalization using the *α*-stable distribution (*α*-stable) [21]. The comparison is carried out by depicting the error bars, which are estimated using 25th and 75th percentile of the mean histogram in the non-specific region, as in [21] (Fig 5). These error bars present the inter-subject intensity variability that is clearly reduced by our normalization methods based on the nonlinear filtering process (FGMM approach) and a linear intensity transformation (MSE approach), as displayed in Fig 5c and 5d. Thus, these methodologies entail a greater degree of homogeneity in the intensity values of the non-specific region, which is in fact the main goal of our intensity normalization procedures.

The difference between the probability distribution of each image denoted by *Q* and the probability distribution of the mean brain image denoted by *P* is evaluated for all subjects before and after intensity normalization using the Kullback-Leibler divergence (KL) [46] which is defined as:

$$D_{KL}\left(P||Q\right)=\sum_{i=1}^{n}\ln(\frac{P(i)}{Q(i)})\;\left(P\left(i\right)\right)$$ (20)

where *n* is a fixed number of bins. The inter-subject distance is calculated quantitatively between these two distributions, both before normalization, for raw images and after normalization, using the proposed and the compared methods. The lowest KL value and the lowest error are obtained (in terms of the standard deviation) by the proposed normalization methods based on linear intensity normalization by MSE optimization and GMM-based image filtering as presented in Table 2. This experimental result suggests that the proposed methods outperform the compared methods, in entailing more intensity homogeneity in the non-specific region.

**Table 2:** Mean Kullback-Leibler distance and standard deviation for all images before and after intensity normalization methods in the non-specific region.

Normalization approach	class	Kullback-Leibler distance
Raw data (spatial normalization)	NCs	0.3021±0.2342
	PS	0.2882±0.2412
	NCs+PS	0.2957±0.2367
BR_*all*_	NCs	0.2549±0.1681
	PS	0.3308±0.2279
	NCs+PS	0.2902±0.2009
**MSE**	NCs	**0.1025±0.0588**
	PS	**0.1196±0.0928**
	NCs+PS	**0.1105±0.0766**
**FGMM**	NCs	**0.0671±0.0588**
	PS	**0.0777±0.0575**
	NCs+PS	**0.0720±0.0582**
*α*-stable	NCs	0.1986±0.1388
	PS	0.2632±0.1808
	NCs+PS	0.2286±0.1623

**Fig 6:**
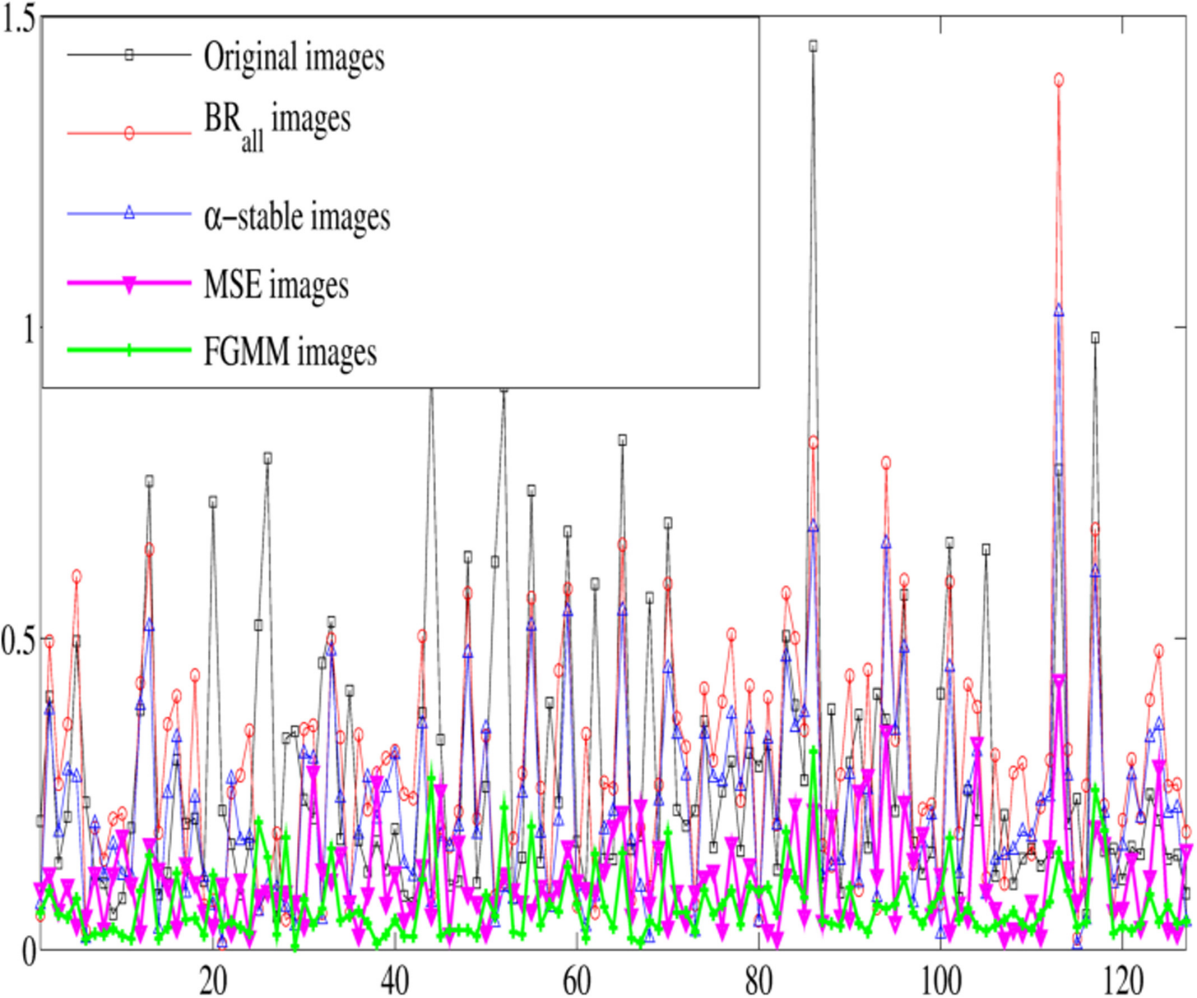
The Kullback-Leiber divergence (KL) of each image before and after intensity normalization.

Fig 6 presents the KL distance for each image in the different datasets (before intensity normalization, for the original images and post-normalization, using the proposed approaches (FGMM and MSE), the standard normalization method (BR_*all*_) and *α*-stable approach). This figure reveals that the inter-subject differences in intensity values in the non-specific region are quantitatively mitigated after the intensity normalization using the proposed methodologies.

**Fig 7:**
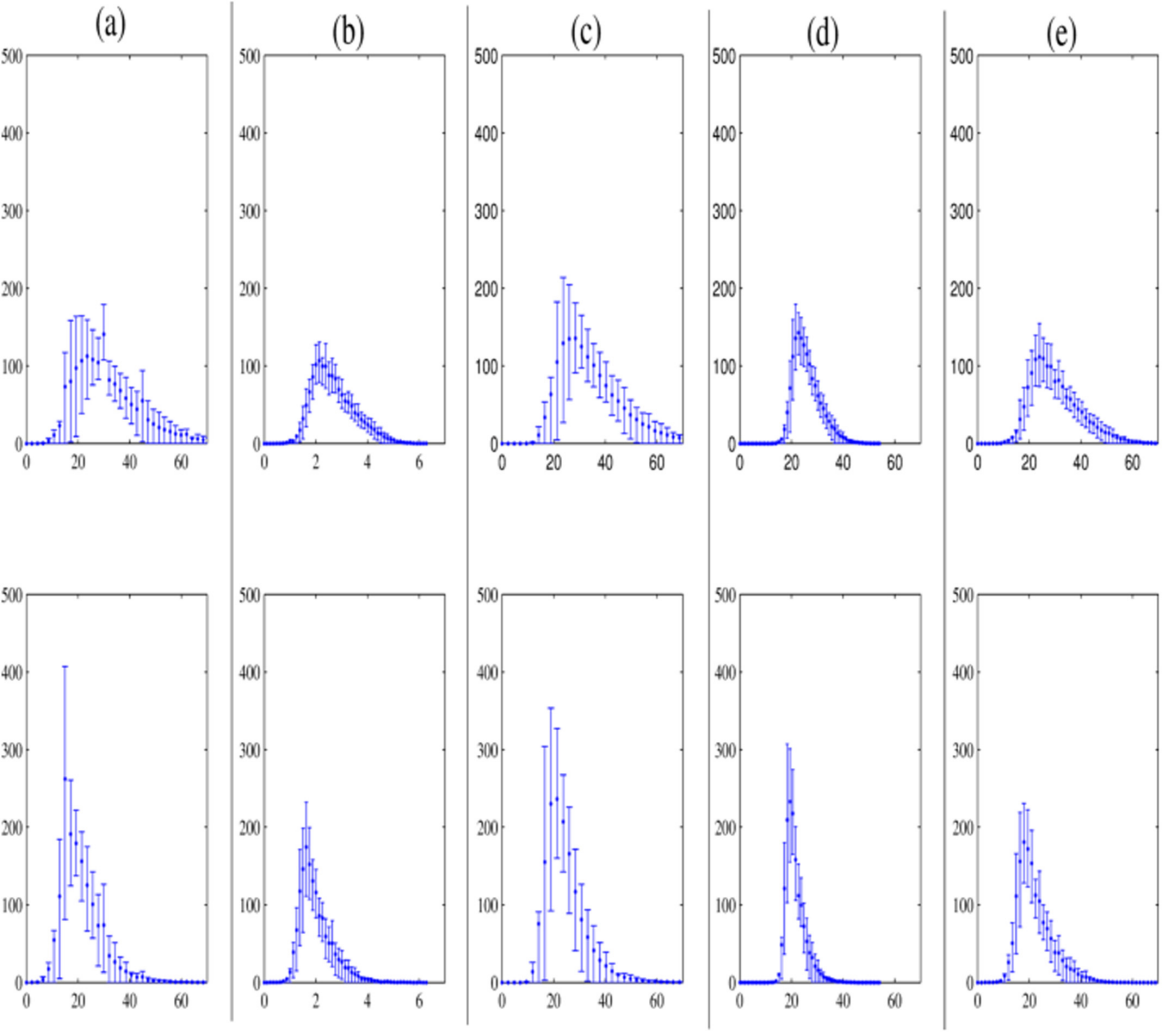
Mean histograms and error bars in the striatum region for 127 DaTSCAN images before and after intensity normalization, keeping separate distributions for the two different classes. The first row is for NC subjects and the second row is for PS subjects. (a): Original DaTSCAN images, (b): BR_*all*_ images, (c): Filtered GMM images, (d): MSE images and (e) *α* stable images. The x-axis represents the intensity. The y-axis indicates the number of voxels with a given level in the striatum region.

Moreover, Fig 7 shows the inter-subject intensity variability in the striatum for NC subjects (first row) and PS subjects (second row) in terms of mean histogram and error bars. Notice that the intensity distributions obtained by our approaches are clearly different in shape and variability for NC and PS subjects (see the first row versus the second row of Fig 7c and 7d. In order to further analyze these results, we propose the following section in which these distributions are considered in classification tasks.

### Quantitative classification performance of Parkinsonism

In order to evaluate the benefits of the proposed intensity normalization methods for PS detection, several experiments were performed on the previously described database. The proposed methods are assessed on the task of discriminating PS from NCs and compared to the BR_*all*_ and *α*-stable techniques. Thus, each intensity normalization procedure leads to a different dataset. For each dataset, the performance of the Support Vector Machines (SVM) [47–50] classifier was studied. Only linear SVM has been used to compute the results, due to the large number of input features to the classifier, to obtain more generalizable results and to avoid the small sample size problem [27]. The accuracy estimation is performed following a Leave-One-Out (LOO) cross-validation strategy. The classifier is trained as many times as the size of the database. In each iteration an image is used for the test and the remaining ones for training. The global accuracy is then calculated as the average of the accuracy achieved in each iteration.

The ^123^ I-ioflupane radiopharmaceutical provides brain images with higher activation in the striatum, a region of high interest for the diagnosis of PS [51]. Fig 4a reveals that most of the activity is gathered in the striatum. However, the images contain a lot of information (a large number of voxels) that is not relevant for the diagnosis of the disorder. For this purpose, a required binary mask is applied to each image for the different datasets in order to select only the high-intensity voxels of the striatum area.

#### Selection of the Region of Interest (ROI)

Once the images of the different datasets are ready for the classification process, the relevant information has to be extracted. Only the voxels that contain relevant information in terms of discrimination ability should be chosen. In the case of Parkinson’s disease, this region is, as previously mentioned, the striatum. For this purpose, we need to apply a binary mask for each image which is computed as follows:

$$m_{i}=\{\begin{array}{ccc} 1 & \text{if} & c_{i}⩾0.45\;max\;c_{i}\\ 0 & \text{otherwise} \end{array}$$ (21)

where *m*_*i*_, *i* = 1…*n* are the *n* voxels of the mask with value (0 or 1), *c*_*i*_, *i* = 1…*n* are the intensity of *n* voxel at position *i* of an intermediate image, c, and *max* *c*_*i*_ is the highest intensity of *c*. The image *c* is computed by using the average of all NCs in each dataset. Applying this mask allows to select the voxels whose intensity is high (compared with the maximum intensity) in healthy subjects. In practice, this is equivalent to select the voxels of the striatum.

Thus, after voxel selection, a set of intensity values is obtained for each subject, arranged in a 1D array. This array is the key data to be processed in classification between NCs and PS.

#### Voxels-as-Features (VAF)

The first applied method to the datasets is the simple VAF approximation [52]. VAF is considered as a baseline in many works like MRI analysis for AD or autism diagnosis, as many studies suggest that this method is, at least, comparable with the visual exam performed by experts [52]. This approximation uses all voxels in each image as a feature vector, which is used as an input to the classifiers. This baseline has been applied to different datasets using the raw images (spatially normalised ^123^ I-ioflupane-SPECT images) and intensity normalized images by the different proposed approaches. The accuracy rate for different normalization methods using the voxel intensity in the striatum is presented in Table 3. In this Table, the use of the proposed intensity normalization approaches on DaTSCAN images show a significant improvement of the performance results over the same VAF approach (91.34% and 90.55%) compared to unnormalized intensity images (raw data), BR_*all*_ and *α*-stable intensity normalization methods, used here as a baseline (87.40%, 88.19% and 86.61%). The behavior of the VAF system with these strategies of preprocessing highlights the benefits of using an intensity normalization, which allow us to compare the striatum area of each image voxel to voxel, assuming that a similar value of intensity in two different subjects corresponds to a similar value of the drug uptake.

**Table 3:** Comparison between the accuracy rates achieved with the proposed intensity normalization methodologies based on VAF approach and linear SVM classifier.

Normalization approach	Accuracy	Sensitivity	Specificity
Raw data	87.40%	86.44%	88.23%
BR_*all*_	88.19%	87.93%	88.40%
**FGMM**	**91.34**%	**90**%	**92.54**%
**MSE**	**90.55**%	**88.52**%	**92.42**%
*α*-stable	86.61%	85%	88.06%

#### Principal Component Analysis (PCA)

The second system tested for the diagnosis of PS for different datasets is based on the PCA feature extraction method [53–56]. As it is shown in Table 4, the proposed methodologies to analyze ^123^ I-ioflupane images provide high accuracy rates for PS diagnosis with peak values over 92.91% for MSE approach and over 92.13% for FGMM. They represent a significant improvement in the incrementation of the accuracy compared with the results obtained by raw images, BR_*all*_ and *α*-stable approaches (89.76%, 90.34% and 88.19%). The improvement in accuracy is due to the ability of PCA to extract patterns explaining the greatest variance in the data. In addition, the dimensionality reduction of PCA is very effective in classification because a higher number of features will easily lead the classifier into the problem of overfitting [54, 55]. However, the VAF approach considers the raw information included in the ROI.

**Table 4:** Comparison between the accuracy rates achieved with the proposed intensity normalization methodologies based on PCA feature extraction method and linear SVM classifier.

Normalization approach	Accuracy	Sensitivity	Specificity
Raw data	89.76%	92.59%	87.67%
BR_*all*_	90.34%	91.22%	90%
**FGMM**	**92.13**%	**91.53**%	**92.65**%
**MSE**	**92.91**%	**94.64**%	**91.55**%
*α*-stable	88.19%	89.29%	87.32%

To sum up, the proposed methods for intensity normalization deserve much attention in the diagnosis of PS. They demonstrate also their ability and robustness to improve computer aided diagnosis performance in DaTSCAN SPECT imaging in combination with SVM classification, as may be seen from Tables 3 and 4 and supported by Fig 7c and 7d.

An important observation is that intensity normalization using MSE and FGMM approaches can prove to be a reasonable trade-off of computational complexity in favor of having an uniform cross-subject distribution of the intensities in the non-specific area and the diagnostic ability of PS detection. The FGMM outperforms the MSE in the sense of entailing intensity normalization in the non-specific region as it leads to a less difference between the images of the same class, and between images of different classes as shown in Table 2. However, MSE approach obtains higher classification results with a peak value of 92.91% for the accuracy and 94.64% for the sensitivity using the PCA system as shown in Table 4. In addition, Fig 7d reveals that the intensity normalization using MSE deeply affects the voxel information in the striatum region which leads to a better sensitivity using the PCA system. Otherwise, FGMM preserves the information in that region. Finally, taking into account the computational load, the MSE approach is less demanding with a computation time of 7 seconds, as can be seen in Eq 18, than the FGMM method which requires a model estimation stage [35]. For our experiments we used machines running Intel® Xeon® processors with 2.67 GHz CPU frequency and having 48 GB of memory. In fact, with this workstation, the computational time for the model estimation stage can reach 1–2 hours of a GMM model with *k* = 64 clusters. However, for the filtering stage, it takes about 281.06 seconds. For the baseline approaches, the computation time varies between 2 and 64.65 seconds.

As a conclusion, both proposed intensity normalization procedures lead to comparable generalization estimations and perform substantially better than the baseline methods.

## Conclusions

The present work evaluates the impact of different intensity normalization methods for the development of a computer aided diagnosis (CAD) system for PS detection based on DaTSCAN image analysis and classification. Two novel alternatives are proposed to establish a comparison between specific/non-specific uptake areas. These methodologies are based on the extraction of intrinsic parameters from ^123^ I-ioflupane-SPECT images without using anatomical information, resulting in two automatic procedures for intensity normalization: GMM-based image filtering and MSE optimization. The FGMM intensity normalization is achieved according to an automatic selection of the occipital cortex by a normalized probability threshold that measures the weight of each kernel or “cluster” on the striatum area, the voxels in the reference region are intensity normalized by removing clusters whose likelihood is negligible. Otherwise, the MSE optimization is performed by a linear transformation of intensity values in each voxel. This method is obtained by minimizing the MSE in the non-specific region between the source and the template image. Further analysis reveals that, post-normalization, the difference between the striatum and the background uptakes is increased. In addition, the inter-subjects intensity differences are quantitatively reduced in the non-specific region utilizing the Kullback-Leibler divergence criteria, and the artifacts and noise affecting the source images are removed. This allows us to guarantee that the differences between the different DaTSCAN brain images (NC and PS subjects) are due only to the uptake of the tracer in the striatum region. These proposed automatic intensity normalization methods demonstrate also its ability and robustness in PS pattern detection as they provide good values of accuracy. These results open the possibility to apply optimized algorithms to improve CAD performance in DaTSCAN SPECT imaging in combination with SVM classification.
