# Correction: Comparison between Different Intensity Normalization Methods in ^123^I-Ioflupane Imaging for the Automatic Detection of Parkinsonism

**DOI:** 10.1371/journal.pone.0135107

**Published:** 2015-07-31

**Authors:** A. Brahim, J. Ramírez, J. M. Górriz, L. Khedher, D. Salas-Gonzalez

The following information is missing from the Funding section: This work was partly supported by the Ministerio de Ciencia e Innovación (MICINN) under the TEC2012-34306 project and the Consejería de Innovación, Ciencia y Empresa (Junta de Andalucía, Spain) under the Excellence Project P11-TIC-7103 and Fondo Europeo de Desarrollo Regional (FEDER).
